# Experimental measurement of the quantum geometric tensor using coupled qubits in diamond

**DOI:** 10.1093/nsr/nwz193

**Published:** 2019-11-27

**Authors:** Min Yu, Pengcheng Yang, Musang Gong, Qingyun Cao, Qiuyu Lu, Haibin Liu, Shaoliang Zhang, Martin B Plenio, Fedor Jelezko, Tomoki Ozawa, Nathan Goldman, Jianming Cai

**Affiliations:** 1 School of Physics, Huazhong University of Science and Technology, Wuhan 430074, China; 2 International Joint Laboratory on Quantum Sensing and Quantum Metrology, Huazhong University of Science and Technology, Wuhan 430074, China; 3 Institut für Quantenoptik & IQST, Albert-Einstein Allee 11, Universität Ulm, D-89081 Ulm, Germany; 4 Institut für Theoretische Physik & IQST, Albert-Einstein Allee 11, Universität Ulm, D-89081 Ulm, Germany; 5 Interdisciplinary Theoretical and Mathematical Sciences Program (iTHEMS), RIKEN, Wako, Saitama 351-0198, Japan; 6 Center for Nonlinear Phenomena and Complex Systems, Université Libre de Bruxelles, B-1050 Brussels, Belgium

**Keywords:** quantum geometry, topological physics, quantum measurement, quantum control, quantum information

## Abstract

Geometry and topology are fundamental concepts, which underlie a wide range of fascinating physical phenomena such as topological states of matter and topological defects. In quantum mechanics, the geometry of quantum states is fully captured by the quantum geometric tensor. Using a qubit formed by an NV center in diamond, we perform the first experimental measurement of the complete quantum geometric tensor. Our approach builds on a strong connection between coherent Rabi oscillations upon parametric modulations and the quantum geometry of the underlying states. We then apply our method to a system of two interacting qubits, by exploiting the coupling between the NV center spin and a neighboring ^13^C nuclear spin. Our results establish coherent dynamical responses as a versatile probe for quantum geometry, and they pave the way for the detection of novel topological phenomena in solid state.

## INTRODUCTION

The quantum geometric tensor (QGT) constitutes a central and ubiquitous concept in quantum mechanics, by providing a geometric structure to the Hilbert space [1–5]. The imaginary part of this tensor corresponds to the well-known Berry curvature [6,7], which acts as an effective ‘electromagnetic’ tensor in parameter space. This geometric quantity, which is formally associated with the parallel transport of wave functions [8], is responsible for striking observable phenomena such as the geometric phase [8], the anomalous Hall effect [9] and topological states of matter [10]. In contrast, the real part of the QGT constitutes the Fubini-Study metric [2,3,5], which defines a notion of distance (a Riemannian metric) in parameter space through the overlap of wavefunctions. This ‘quantum metric’, which is intimately related to quantum fluctuations and dissipative responses of the system [2,5,11,12], has been shown to play an important role in various contexts, including quantum phase transitions [13], open quantum systems [14], orbital magnetism [15,16], localization in insulators [11], semiclassical dynamics [17,18], excitonic Lamb-shifts in transition-metal dichalcogenides [19], superfluidity in flat bands [20] and topological matter [21,22]. In the context of quantum information, the quantum metric is equivalent to the quantum Fisher information, which is a witness for multipartite entanglement [23].

Various manifestations of the QGT have been observed in experiments, using very different physical platforms and probes. On the one hand, the local Berry curvature has been detected in ultracold atomic gases [24–26], coupled optical fibers [27], and solids [28,29]. On the other hand, a first manifestation of the quantum metric—the so-called Wannier-spread functional of Bloch bands [30]—was recently measured in cold atoms [31], based on the proposal [32]; see [12,33–35] for other proposals to detect quantum geometry. Nevertheless, direct and systematic measurement of the complete QGT has never been performed.

Here, we report on the first experimental measurement of the complete QGT, using a qubit formed by an NV center spin in diamond. Following the proposal of [32], we exploit the relation between the QGT and the response of quantum systems upon parametric modulations to map out the full Fubini-Study metric as well as the local Berry curvature of the underlying quantum states. We then apply our method to a system of two interacting qubits, obtained by coupling the NV center spin to a nearby ^13^C nuclear spin. Our results not only enforce the deep connections between out-of-equilibrium dynamics and quantum geometry [36–44], but also reveal a universal tool for detection of geometric and topological properties in quantum systems.

## DETECTING THE QGT THROUGH RABI OSCILLATION

We start by considering the Hamiltonian $H( {\boldsymbol{\lambda }} )$ of a generic discrete quantum system, which depends on a set of dimensionless parameters $ {\boldsymbol{\lambda}} = \ ( {{\lambda _1},\ {\lambda _2},\ \cdots ,\ {\lambda _N}} )$, where $N\ $is the dimension of parameter space. For a single qubit, the relevant parameter space corresponds to the two-dimensional Bloch sphere. Defining the eigenstates and eigenvalues of this generic Hamiltonian, $H( {\boldsymbol{\lambda }} )\ | {n( {\boldsymbol{\lambda }} )}\rangle = {\epsilon _n}\ ( {\boldsymbol{\lambda }} )| {n( {\boldsymbol{\lambda }} )}\rangle ,$ a geometric structure emerges upon projecting the dynamics onto a single (non-degenerate) band ${\epsilon _n}( {\boldsymbol{\lambda }} ).$ The resulting quantum geometry is captured by the QGT, which is defined as [7]

(1) \begin{equation*}\chi _{\mu \nu }^{\left( n \right)} = \left\langle {{\partial _\mu }n({\boldsymbol{\lambda }})\big| {{\rm{(}}1 - {\big|}n({\boldsymbol{\lambda }})}} \right\rangle \left\langle {n({\boldsymbol{\lambda }})\big|)\big|{\partial _\nu }n({\boldsymbol{\lambda }})} \right\rangle .\end{equation*}

For simplicity, hereafter we denote ${\partial _\mu } \equiv {\partial _{{\lambda _\mu }}}$. The real part $Re\ ( {{\chi _{\mu \nu }}} ) = {g_{\mu \nu }}\ $ is the Fubini-Study metric, which introduces a notion of distance in parameter space, while the imaginary part $Im\ ( {{\chi _{\mu \nu }}} ) = - {F_{\mu \nu }}/2$ is related to the Berry curvature$\ {F_{\mu \nu }}$ responsible for the Berry phase. It is useful to express the QGT in the form

(2) \begin{eqnarray*} &&\chi _{\mu \nu }^{\left( n \right)} = {\sum _{m \ne n}} \nonumber\\ &&\times\frac{{\left\langle {n\hskip-1pt\left( {\boldsymbol{\lambda }} \right)\hskip-2pt\left|\hskip-0pt {{\partial _\mu }H\left( {\boldsymbol{\lambda }} \right)} \hskip-1pt\right|\hskip-1pt m\left( {\boldsymbol{\lambda }} \right)} \right\rangle \left\langle {m\left( {\boldsymbol{\lambda }} \right)\hskip-2pt\left|\hskip-0pt {{\partial _\nu }H\left( {\boldsymbol{\lambda }} \right)} \hskip-0pt\right|\hskip-1pt n\left( {\boldsymbol{\lambda }} \right)} \right\rangle }}{{{{\left( {{\epsilon _m}\left( {\boldsymbol{\lambda }} \right) - {\epsilon _n}\left( {\boldsymbol{\lambda }} \right)} \right)}^2}}}, \nonumber\\ \end{eqnarray*}

so as to highlight the relation between this geometric quantity and the coupling matrix elements connecting the eigenstates $ {|n( {\boldsymbol{\lambda }} )} \rangle $ and $|m {( {\boldsymbol{\lambda }} )} \rangle $ upon a parametric modulation [32],

(3) \begin{equation*}{{\rm{\Omega }}_{n \leftrightarrow m}}\left( {\boldsymbol{\lambda }} \right) \propto \left\langle {m\left( {\boldsymbol{\lambda }} \right)\left| {{\partial _\mu }H\left( {\boldsymbol{\lambda }} \right)} \right|n\left( {\boldsymbol{\lambda }} \right)} \right\rangle .\end{equation*}

## EXPERIMENTAL SETUP

In our experiment, we first perform a full quantum-geometric measurement using a two-level system, as described by the general Hamiltonian

(4) \begin{equation*}H\left( {\theta ,\varphi } \right)\ = \frac{A}{2}\ \left( {\begin{array}{@{}*{2}{c}@{}} {\cos \theta }&{\sin \theta {e^{ - i\varphi }}}\\ {\sin \theta {e^{i\varphi }}}&{ - \cos \theta } \end{array}} \right),\end{equation*}

where the angles $( {\theta ,\ \varphi } )$ form the relevant parameter space (the Bloch sphere). Considering the low-energy dressed state, the components of the QGT read ${g_{\theta \theta }} = \frac{1}{4}\ ,\ \ \ {g_{\varphi \varphi }} = \frac{1}{4}\ {\sin ^2}\theta ,\ \ \ {g_{\theta \varphi }} = \ 0,\ \ \ {F_{\theta \varphi }} = \sin \theta /2.$ These components fully characterize the underlying quantum geometry: the quantum metric $g$ corresponds to the natural metric of a sphere ${S^2}$, embedded in ${R^3}$ with fixed radius $R = \frac{1}{2}$, while the Berry curvature ${F_{\theta \varphi }}$ corresponds to the ‘magnetic’ field of a fictitious Dirac monopole located at the center of that sphere [22].

**Figure 1.**
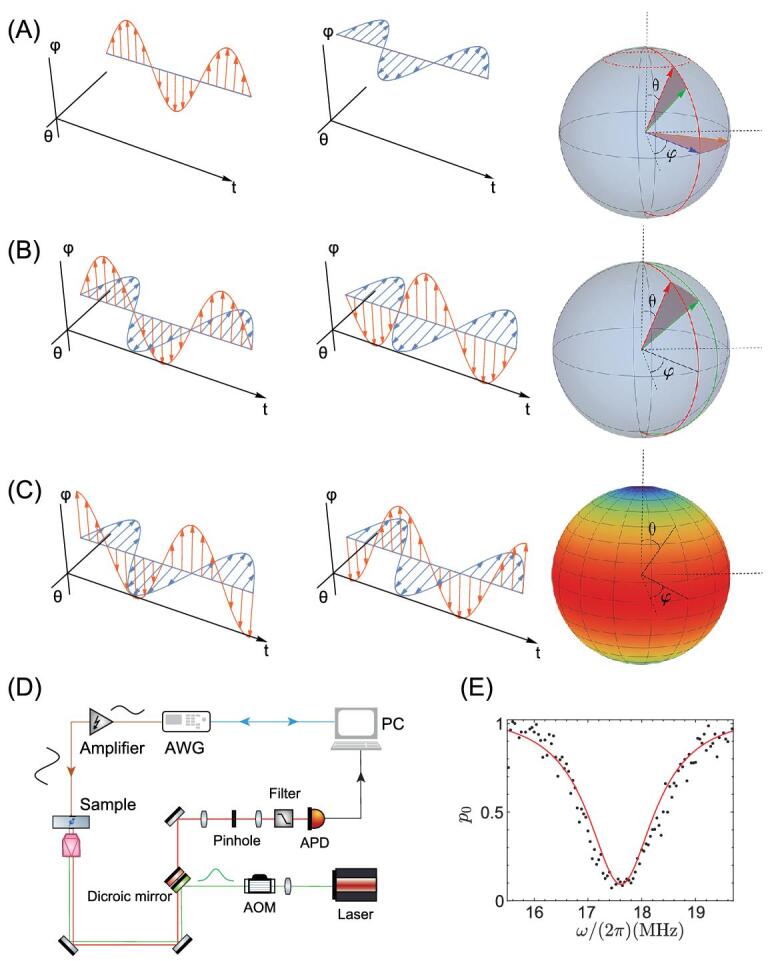
Probing quantum geometry through coherent responses on parametric modulations. (A–C) show different types of parametric modulations $( {{\theta _t},\ {\varphi _t}} )$, including (A, B) linear parametric modulation ${\theta _t} = {\theta _0} + {a_\theta }\sin ( {\omega t} ),\ {\varphi _t} = {\varphi _0} + {a_\varphi }\sin ( {\omega t} )$ for measurement of the diagonal [off-diagonal] element of the Fubini-Study metric with ${a_\theta } = 0$ or ${a_\varphi } = 0$ (A) [${a_\theta } = \pm {a_\varphi } \ne 0$ (B)]; (C) elliptical parametric modulation for the measurement of the local Berry curvature (as indicated by color map) with ${\theta _t} = {\theta _0}\ + {a_\theta }\sin ( {\omega t} )$ and ${\varphi _t} = {\varphi _0}\ \pm {a_\varphi }\cos ( {\omega t} )$. (D) Experimental setup used for the quantum-geometric measurement, based on an NV center spin in diamond. A green laser pulse polarizes the NV center spin into the $|\ {m_s} = 0\rangle $ state. The engineered microwave created from an arbitrary waveform generator (Tektronix AWG 70002A, 16GS/s) is amplified before being delivered to the sample and coherently drives the NV center spin. The NV center spin state is detected by an APD via spin-dependent fluorescence. (E) An example of parametric-modulation resonance measurement. The probability that the NV center spin remains in the initial eigenstate at time $T = 400\ {\rm{ns\ }}$as a function of the modulation frequency, for a linear parametric modulation ${\theta _t} = {\theta _0} + {a_\theta }\sin ( {\omega t} ),\ {\varphi _t} = {\varphi _0}\ $ with $( {{\theta _0},\ \ {\varphi _0}} ) = ( {\frac{{5\pi }}{6},\ 0} )\ $ and $\ {a_\theta } = 0.1$.

The experimental setup is sketched in Fig. 1D. The two-level system in Eq. (4) is obtained from a single nitrogen-vacancy (NV) center in an electronic grade diamond. We apply a magnetic field ${B_z} = \ 509\,G$ along the NV axis to lift the degeneracy of the states ${m_s} = \ \pm 1.$ A two-level system is supported by the spin sublevels ${m_s} = \ 0$ and ${m_s} = - 1.$ We first prepare the system in the eigenstate of the Hamiltonian $H( {{\theta _0},\ {\varphi _0}} )$, i.e. $n( {{\theta _0},\ {\varphi _0}} )\rangle = \cos \frac{{{\theta _0}}}{2}\ | - 1\rangle + \sin \frac{{{\theta _0}}}{2}{e^{i{\varphi _0}}}|0\rangle $. This is achieved by first applying a 532 nm green laser pulse to initialize the NV center spin in the ${m_s} = 0\ $state. A subsequent microwave pulse ${H_i}( t ) = {\rm{\Omega }}\sin ( {{\omega _0}t + {\varphi _0}} ){\sigma _x},$ applied over a duration ${t_{{\theta _0}}} = \frac{{{\theta _0}}}{\Omega }$, rotates the NV center spin around the axis $\hat{n}( {{\varphi _0}} ) = ( {\cos {\varphi _0},\ \sin {\varphi _0},0} )$ by an angle ${\theta _0}$. The initial state preparation is verified by a spin-locking type experiment, which confirms that the NV spin is prepared in the eigenstate of $H( {{\theta _0},\ {\varphi _0}} )$ [see supplementary data].

The precise control over the AWG allows us to engineer the microwave driving field with accurate amplitude and phase modulation. This leads to implementation of the generic two-level system

(5) \begin{equation*}H\ \left( t \right) = \frac{{{\omega _0}}}{2}\ {\sigma _z} + V\left( t \right){\sigma _x},\end{equation*}

where$\ V\ ( t ) = ( {A\sin {\theta _t}} )\cos [ {{\omega _0}t - f( t ) + {\varphi _t}} ]$. In the experiment, we calibrate the driving amplitude in the Hamiltonian [Eq. (5)] with the output power of the AWG by measuring the Rabi frequency of the NV center spin [see supplementary data]. The amplitude modulation $A\sin {\theta _t}$ and the phase modulation $ - f( t ) + {\varphi _t}$ are synthesized by waveform programming in the AWG. The additional phase control function has the form $f\ ( t ) = \ A\int_{0}^{t}{{\cos {\theta _\tau }d\tau \cong A\cos {\theta _0}{J_0}( {{a_\theta }} )t - ({4A\sin {\theta _0}/\omega })}}$${{{J_1}( {{a_\theta }} ){{\sin }^2}( {\omega t/2})}}$, where ${J_{0,1}}$ are the zeroth and first order Bessel functions of the first kind, respectively [see supplementary data]. Taking the limit$\ {\omega _0} \gg A$, such an engineered microwave driving field allows us to realize the effective Hamiltonian in Eq. (4) with the designed parametric modulation [see supplementary data]:

(6) \begin{eqnarray*} &&{H_{eff}}\left( t \right) \cong \frac{A}{2} \nonumber\\ &&\quad \times\, \big[ \cos {\theta _t}{\sigma _z} + \sin {\theta _t}\big( {\cos {\varphi _t}{\sigma _x} + \sin {\varphi _t}{\sigma _y}} \big) \big]. \nonumber\\ \end{eqnarray*}

The parametric modulation drives a coherent transition between the eigenstates of $H( {{\theta _0},\ {\varphi _0}} )$, which is detected by rotating the NV center spin around the axis $\hat{n}( {{\varphi _0}} )$ by an angle $2\pi - {\theta _0}$. This rotation maps the eigenstates of $H( {{\theta _0},\ {\varphi _0}} )$ back to the NV center spin state $|0\rangle $ and $| - 1\rangle $, which is then measured by spin-dependent fluorescence.

## EXPERIMENTAL RESULTS

In the experiment, we implement two types of modulations [32]: (a) a ‘linear’ modulation ${\theta _t} = {\theta _0}\ + {a_\theta }\sin ( {\omega t} )$, ${\varphi _t} = {\varphi _0}\ + {a_\varphi }\sin ( {\omega t} )$; (b) an ‘elliptical’ modulation ${\theta _t} = {\theta _0}\ + {a_\theta }\sin ( {\omega t} )$, ${\varphi _t} = {\varphi _0}\ + {a_\varphi }\cos ( {\omega t} )$; see Fig. 1A–C. Setting ${a_\theta },\ {a_\varphi } \ll 1$, the time-dependent Hamiltonian can be expressed as

(7) \begin{equation*}\begin{array}{@{}*{3}{l}@{}} {H( {{\theta _t},{\varphi _t}})}& \cong &{H( {{\theta _0},{\varphi _0}}) + {a_\theta }( {{\partial _\theta }H})\sin( {\omega t})}\\ {}&{}&{ + \ {a_\varphi }( {{\partial _\varphi }H} )\sin( {\omega t}):\ linear}\\ {}&{}&{ + \ {a_\varphi }( {{\partial _\varphi }H})\cos ( {\omega t}):\ elliptical.} \end{array}\end{equation*}

**Figure 2.**
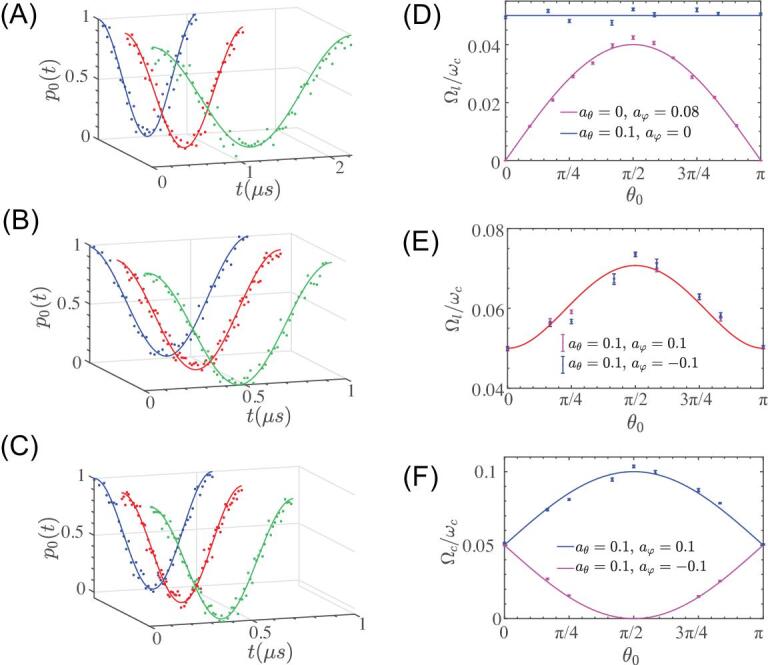
Coherent transitions induced by parametric modulations. (A, B) Resonant oscillation under a linear parametric modulation with ${a_\theta } = 0$, ${a_\varphi } = 0.08$ (A) and ${a_\theta } = 0.1$, ${a_\varphi } = 0.1$ (B). (C) Resonant oscillation under an elliptical parametric modulation with $\ {a_\theta } = 0.1$, $\ {a_\varphi } = 0.1$. The other experimental parameters are: (A) ${\omega _c} = ( {2\pi } )20.98\ {\rm{MHz\ }}$(${\theta _0} = \frac{\pi }{6}$, green), $( {2\pi } )21.61\ {\rm{MHz\ }}$(${\theta _0} = \frac{\pi }{3}$, red), $( {2\pi } )20.73$$ {\rm{MHz\ }} ({\theta _0} = \frac{\pi }{2}$, blue); (B) ${\omega _c} = ( {2\pi } )19.11\ {\rm{MHz\ }}$(${\theta _0} = \frac{\pi }{6}$, green), $( {2\pi } )17.8\ {\rm{MHz\ }}({\theta _0} = \frac{{5\pi }}{{12}}$, red), $( {2\pi } )16.72\ {\rm{MHz\ }}$(${\theta _0} = \frac{\pi }{2}$, blue); (C) ${\omega _c} = \ ( {2\pi } )19.11\ {\rm{MHz\ }}$(${\theta _0} = \frac{\pi }{6}$, green),$\ ( {2\pi } )17.8\ {\rm{MHz\ }}$(${\theta _0} = \frac{{5\pi }}{{12}}$, red), $( {2\pi } )16.72\ {\rm{MHz\ }}$(${\theta _0} = \frac{\pi }{2}$, blue). (D–F) Rabi frequency of resonant coherent transitions upon parametric modulations (in the unit of resonant frequency$\ {\omega _c}$), as a function of the parameter ${\theta _0}$, for linear (D, E) and elliptical (F) parametric modulations. The curves show theoretical predictions. In (A–F), we set the parameter ${\varphi _0} = \ $0.

After preparing the NV center spin in the eigenstate $|n( {{\theta _0},\ {\varphi _0}} )\rangle $ of the Hamiltonian $H( {{\theta _0},\ {\varphi _0}} )$, we apply the engineered microwave driving field with parametric modulation [see Eq. (5)] and fix the time duration $T$. We sweep the parametric modulation frequency $\omega $, and measure the probability ${p_0}( T )$ that the NV spin remains in the initial eigenstate $|n( {{\theta _0},\ {\varphi _0}} )\rangle $. In Fig. 1E, we show an example of such a parametric-modulation resonance measurement; see supplementary data for the experimental data using other types of modulations. The results indicate that a coherent transition between the eigenstates becomes resonant when $\omega \cong A \equiv {\omega _c}$. We then measure the resonant coherent oscillation upon parametric modulation with $\omega $ =${\omega _c}$, as shown in Fig. 2A–C. The observed Rabi frequencies under resonant parametric modulations, which reveal the information about the coupling matrix elements connecting the eigenstates [see Eq. (3)] upon parametric modulation, are shown in Fig. 2D–F. The experimental results allow us to determine the quantum geometry of the prepared dressed states precisely.

**Figure 3.**
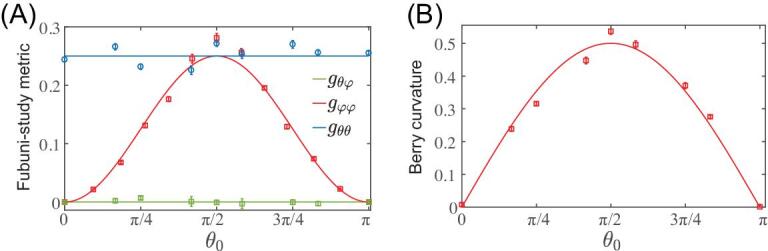
Extraction of the complete quantum geometric tensor. (A) The measured Fubini-Study metric, compared with the theoretical predictions ${g_{\theta \varphi }} = 0$ (green curve), ${g_{\varphi \varphi }} = {\rm{si}}{{\rm{n}}^2}\ {\theta _0}/4$ (red curve) and ${g_{\theta \theta }} = 1/4$ (blue curve). (B) The measured local Berry curvature ${F_{\theta \varphi }}$ is compared with the theoretical prediction ${F_{\theta \varphi }} = \sin {\theta _0}/2\ $. The experimental parameters are the same as in Fig. 2.

As a central result, we show in Fig. 3 the experimental extraction of the full QGT, based on Rabi-oscillation measurements. This provides a first demonstration that coherent responses upon parametric modulations can be used as a powerful tool to access the complete geometry of a discrete quantum system. We point out that the present quantum-geometry measurement is based on coherent dynamic responses upon periodic driving, and in this sense, it does not rely on any adiabaticity constraints (i.e. small modulation velocity [36,37]). It should be noted, however, that this method uses small modulation amplitudes, and hence small Rabi frequencies, which require systems exhibiting long coherence times. The agreement between the experiment results and the theoretical predictions can be improved by increasing the measurement time, which allows for better determination of the oscillation frequency. Furthermore, in contrast with the excitation-rate measurement of [31,32,41], the QGT is extracted from Rabi oscillations [39], where the initial state is recovered after each Rabi period; in principle, this allows for detection of geometry and topology through a non-destructive measurement.

Besides, our quantum-geometry measurement can also be used to characterize the topology of the underlying system. For this analysis, we extend the Hamiltonian to the form

**Figure 4.**
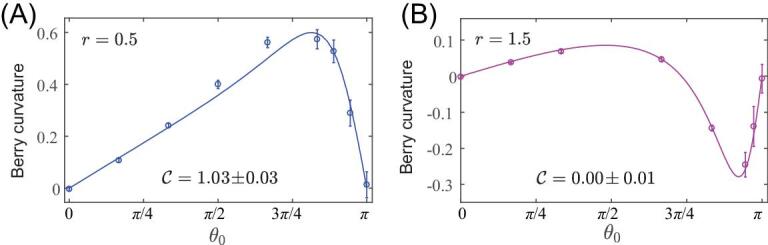
Berry curvature measurement across the topological transition. (A, B) show the measured local Berry curvature ${F_{\theta \varphi }}$ for the Hamiltonian in Eq. (8), which describes a Dirac monopole located inside (A, $r = 0.5$) and outside (B, $r = 1.5$) the Bloch sphere. The curves represent the corresponding theoretical values. The Chern number extracted from the data is indicated in both panels.

(8) \begin{equation*}H\ \left( {\theta ,\varphi } \right) = \frac{A}{2}\ \left( {\begin{array}{@{}*{2}{c}@{}} {\cos \theta + r}&{\sin \theta {e^{ - i\varphi }}}\\ {\sin \theta {e^{i\varphi }}}&{ - \cos \theta - r} \end{array}} \right),\end{equation*}

where $r$ is a tunable parameter. As for Eq. (4), the geometry of the Hamiltonian in Eq. (8) is that of a fictitious monopole located close to a sphere ${S^2}$, whose position in parametric space depends on the additional parameter $r$. The topology of the system then relies on whether this fictitious monopole is located inside the sphere or not, as captured by the Chern number $C = \frac{1}{{2\pi }}\ \mathop \smallint \nolimits_{{S^2}} {F_{\theta \varphi }}d\theta d\varphi $ [13]. Figure 4 shows the Berry curvature measurement in two distinct topological phases. In the non-trivial regime, the Chern number can equally be determined from the metric${\rm{\ }}C = \frac{1}{{2\pi }}\ \mathop \smallint \nolimits_{{S^2}} ( {2\sqrt {\bar{g}} } )d\theta d\varphi = \frac{1}{{2\pi }}\ \mathop \smallint \nolimits_{{S^2}} | {{F_{\theta \varphi }}} |d\theta d\varphi $, where $\bar{g} = {g_{\theta \theta }}\ {g_{\varphi \varphi }} - {( {{g_{\theta \varphi }}} )^2}$ is the determinant of the QGT [22]. Altogether, these results indicate that topology can indeed be finely analyzed based on our geometric-detection scheme.

## APPLICATION TO INTERACTING QUBITS

As a second application, we further extend our experiment to extract the QGT of an interacting two-qubit system. The interacting two-qubit system is formed by an NV center electron spin coupled to a ^13^C nuclear spin located in the vicinity of the NV center. We determine the strength of the corresponding spin-spin interactions using a pulsed optically detected magnetic resonance experiment; we obtain the interaction parameters: ${A_x} \approx 2.79$ MHz and ${A_z} \approx 11.832$ MHz [see Eq. (9) below]. By engineering microwave driving fields with designed frequency and phase, we obtain the following effective Hamiltonian

(9) \begin{eqnarray*} && {H_{rot}}\ \left( {\theta ,\varphi } \right) = \frac{{{{\rm{\Omega }}_{mw}}}}{2} \nonumber\\ &&\quad \times\, \left[ {\cos \theta {\sigma _z} + \sin \theta \left( {\cos \varphi {\sigma _x} + \sin \varphi {\sigma _y}} \right)} \right] \nonumber\\ &&\quad +\, \left( {\frac{{{\gamma _n}{B_\parallel }}}{2} - \frac{{{A_z}}}{4}} \right){\tau _z} - \frac{{{A_x}}}{4}{\tau _x} \nonumber\\ &&\quad -\, \frac{{{A_z}}}{4}{\sigma _z} \otimes {\tau _z} - \frac{{{A_x}}}{4}{\sigma _z} \otimes {\tau _x}, \end{eqnarray*}

where ${\rm{\sigma }}$ and ${\rm{\tau }}$ are Pauli operators associated with the first and second qubits, respectively. Henceforth, we denote the eigenstates of the Hamiltonian in Eq. (9) as $| {{{\rm{\Psi }}_1}}\rangle ,\ | {{{\rm{\Psi }}_2}} \rangle,\ | {{{\rm{\Psi }}_3}} \rangle,\ | {{{\rm{\Psi }}_4}}\rangle ,$ according to their ordered eigenenergies ${\epsilon _1} < {\epsilon _2} < {\epsilon _3} < {\epsilon _4}.$

The competition between the local term (${{\rm{\Omega }}_{{\rm{mw}}}}$) and the spin-spin interaction in the Hamiltonian Eq. (9) leads to a rich topological phase diagram. In the regime ${{\rm{\Omega }}_{{\rm{mw}}}} \gg {\rm{\Omega }}_{mw}^{( {{c_1}} )}$, where

**Figure 5.**
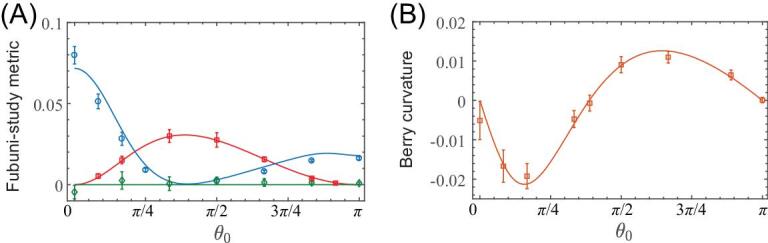
Quantum geometry of an interacting two-qubit system. (A) The measured Fubini-Study metric, compared with the theoretical predictions: ${g_{\theta \varphi }}$ (green curve), ${g_{\varphi \varphi }}$ (red curve) and ${g_{\theta \theta }}$ (blue curve). (B) The measured local Berry curvature ${F_{\theta \varphi }}$ is compared with the theoretical prediction (curve). The amplitude of the driving field [see Eq. (9)] is ${{\rm{\Omega }}_{mw}} = \ 2.13\ {\rm{MHz}}.$ The Chern number estimated from the integral of the Berry curvature is ${\rm C} = 0.00 \pm 0.01$, which is in agreement with the prediction (${\rm C} = 0$) in this strongly interacting regime.

(10) \begin{equation*}{\rm{\Omega }}_{mw}^{\left( {{c_1}} \right)} = \frac{1}{2}\ \left[ { - {\gamma _n}{B_\parallel } + \sqrt {{{\left( {{\gamma _n}{B_\parallel } - {A_z}} \right)}^2} + A_x^2} } \right],\end{equation*}

the spin-spin interaction becomes less significant and we thus recover the topological properties of the two-level system, for which the Chern number is ${\rm{C\ }} = {\rm{\ }}1$ in the eigenstate $|{{\rm{\Psi }}_3}\rangle $ (see the measurements described in the previous section); note that the other eigenstates exhibit similar behaviors. The spin-spin interaction eventually dominates upon decreasing the value of the local parameter; below the critical value, ${{\rm{\Omega }}_{{\rm{mw}}}} < {\rm{\Omega }}_{mw}^{( {{c_1}} )}$, the Chern number of the eigenstate changes from ${\rm{C}} = 1$ to ${\rm{C}} = 0$, which can be seen as a drastic effect of the spin-spin interaction. This vanishing of the Chern number in the strongly interacting regime is clearly captured by our QGT measurement, as reported in Fig. 5. These results demonstrate the measurement of both the Fubini-Study metric and the Berry curvature deep in the interacting regime, and show excellent agreement with theoretical predictions [see supplementary data].

As previously noted, the QGT contains information regarding the entanglement properties of interacting systems, through the concept of quantum Fisher information [23]. As an interesting perspective, our detection method could be applied to more complex interacting systems in view of revealing their quantum fluctuations and entanglement properties.

## CONCLUSION

To summarize, we have experimentally demonstrated a powerful connection between the quantum geometric tensor and the coherent dynamic response of a quantum system upon a parametric drive. Based on this fundamental relation, we first extracted the complete QGT, including all the components of the Fubini-Study metric and those of the local Berry curvature, by driving Rabi oscillations in a single qubit. These measurements clearly revealed the topological (monopole-type) structure associated with this simple setting. We point out that this method is readily applicable to observe other intriguing topological defects, such as tensor monopoles defined in 4D parameter spaces [22]. Furthermore, we have applied this detection method to an interacting two-qubit system, which suggests potential applications to many-body quantum systems with geometric features [12,32,45]. Altogether, our results demonstrate that coherent dynamic responses can serve as a powerful tool to access the geometric and topological properties of quantum systems and open a way to explore the fundamental role of the QGT in various scenarios, ranging from many-body systems to open quantum systems.

## Note added

Materials and methods are available as supplementary material. Two other experimental measurements of the QGT were reported after the completion of our work [46], in polaritons [47] and superconducting qubits [48].

## Supplementary Material

nwz193_Supplemental_File
