# Supplementary material for: Experimental measurement of the quantum geometric tensor using coupled qubits in diamond
Source: Natl Sci Rev. 2019 Nov 27;7(2):254–60. doi: 10.1093/nsr/nwz193 (PMC8288884; doi:10.1093/nsr/nwz193)
Supplement: nwz193_Supplemental_File [file nwz193_supplemental_file.pdf]

Supplementary Information for: "Experimental measurement of the quantum geometric tensor using coupled qubits in diamond"

A. CONNECTION BETWEEN QUANTUM GEOMETRY AND RABI OSCILLATION

A.1 Parametric modulation induced coherent transition for discrete quantum systems

In the main text, we experimentally demonstrate the connection between parametric modulation induced coherent transition and quantum geometric tensor for discrete quantum systems. Here, we provide a detailed analysis on such a fundamental connection following Ref.[1]. Consider a discrete quantum system with a general Hamiltonian $\mathcal{H}(\boldsymbol{\lambda})$ which depends on a set of dimensionless parameters $\boldsymbol{\lambda} = (\lambda_1, \lambda_2, \cdots, \lambda_N)$ where N is the dimension of parameter space, the eigenstates are given as follows

$$\mathcal{H}(\boldsymbol{\lambda})|n(\boldsymbol{\lambda})\rangle = \epsilon_n(\boldsymbol{\lambda})|n(\boldsymbol{\lambda})\rangle. \tag{1}$$

If the Hamiltonian has no energy degeneracy, the definition of quantum geometric tensor (QGT) is [2]

$$\chi_{\mu\nu} = \left\langle \partial_\mu n(\boldsymbol{\lambda}) \middle| \left(1 - |n(\boldsymbol{\lambda})\rangle\langle n(\boldsymbol{\lambda})|\right) \middle| \partial_\nu n(\boldsymbol{\lambda}) \right\rangle. \tag{2}$$

The real part of QGT $\mathrm{Re}(\chi_{\mu\nu}) = g_{\mu\nu}$ is the Fubini-Study metric that quantifies the distance between nearby states $|n(\boldsymbol{\lambda})\rangle$ and $|n(\boldsymbol{\lambda} + d\boldsymbol{\lambda})\rangle$ on parametric manifold, the imaginary part $\mathrm{Im}(\chi_{\mu\nu}) = -\mathcal{F}_{\mu\nu}(\boldsymbol{\lambda})/2$ where $\mathcal{F}_{\mu\nu}(\boldsymbol{\lambda})$ is the local Berry curvature, which is responsible for the geometric (Berry) phase. The QGT is shown to connect with the coherent response on parametric modulation [1]. In the main text, we consider two types of parametric modulation $\left[\lambda_\mu(t), \lambda_\nu(t)\right]$: (a) the linear parametric modulation with $\lambda_\mu(t) = \lambda_\mu^0 + a_\mu \sin(\omega t)$, $\lambda_\nu(t) = \lambda_\nu^0 + a_\nu \sin(\omega t)$; (b) the elliptical parametric modulation with $\lambda_\mu(t) = \lambda_\mu^0 + a_\mu \sin(\omega t)$, $\lambda_\nu(t) = \lambda_\nu^0 + a_\nu \cos(\omega t)$.

As a specific example of linear parametric modulation with $a_\mu \neq 0$ and $a_\nu = 0$, when considering weak parametric modulation i.e. $a_\mu \ll 1$, the time-dependent Hamiltonian can be expanded as

$$\mathcal{H}[\boldsymbol{\lambda}(t)] = \mathcal{H}(\boldsymbol{\lambda}^0) + a_\mu \left[\partial_\mu \mathcal{H}(\boldsymbol{\lambda}^0)\right] \sin(\omega t). \tag{3}$$

Assume that the initial state is prepared on the ground state $|n(\boldsymbol{\lambda}^0)\rangle$, it will be excited onto the higher energy levels by the time-dependent Hamiltonian. Since the Hamiltonian is periodic, the problem can be solved by Floquet theorem. But if the frequency of modulation is on resonance with the energy detuning between $|n(\boldsymbol{\lambda}^0)\rangle$ and $|m(\boldsymbol{\lambda}^0)\rangle$ namely $\hbar\omega = \hbar\omega_{n\leftrightarrow m} \equiv |\epsilon_m(\boldsymbol{\lambda}) - \epsilon_n(\boldsymbol{\lambda})|$, the Floquet Hamiltonian can be simplified as a two-level Hamiltonian in the Hilbert subspace spanned by $\{|n(\boldsymbol{\lambda}^0)\rangle, |m(\boldsymbol{\lambda}^0)\rangle\}$ as follows

$$\mathcal{H}_{\mathrm{rot}}(\boldsymbol{\lambda}^0) = \begin{pmatrix} \epsilon_m(\boldsymbol{\lambda}) - \hbar\omega & \Omega_{n\leftrightarrow m}(\boldsymbol{\lambda}) \\ \Omega^*_{n\leftrightarrow m}(\boldsymbol{\lambda}) & \epsilon_n(\boldsymbol{\lambda}) \end{pmatrix}, \tag{4}$$

where

$$\Omega_{n\leftrightarrow m}(\boldsymbol{\lambda}) = \frac{a_\mu}{2}\langle m(\boldsymbol{\lambda})|\partial_\mu \mathcal{H}(\boldsymbol{\lambda})|n(\boldsymbol{\lambda})\rangle, \tag{5}$$

with the following relation as

$$\begin{aligned} &\left|\langle m(\boldsymbol{\lambda})|\partial_\mu \mathcal{H}(\boldsymbol{\lambda})|n(\boldsymbol{\lambda})\rangle\right|^2 \\ &= [\epsilon_m(\boldsymbol{\lambda}) - \epsilon_n(\boldsymbol{\lambda})]^2 \langle \partial_\mu n(\boldsymbol{\lambda})|m(\boldsymbol{\lambda})\rangle\langle m(\boldsymbol{\lambda})|\partial_\mu n(\boldsymbol{\lambda})\rangle. \end{aligned} \tag{6}$$

According to the definition of QGT, it can be seen that the diagonal element of the Fubini-Study metric can be expressed as

follows

$$g_{\mu\mu}(\boldsymbol{\lambda}) = \left\langle \partial_\mu n(\boldsymbol{\lambda}) \middle| (1 - |n(\boldsymbol{\lambda})\rangle\langle n(\boldsymbol{\lambda})|) \middle| \partial_\mu n(\boldsymbol{\lambda}) \right\rangle \tag{7}$$

$$= \sum_{m\neq n} \left\langle \partial_\mu n(\boldsymbol{\lambda}) | m(\boldsymbol{\lambda}) \right\rangle \langle m(\boldsymbol{\lambda}) | \partial_\mu n(\boldsymbol{\lambda}) \rangle \tag{8}$$

$$= \sum_{m\neq n} \frac{|\langle m(\boldsymbol{\lambda})|\partial_\mu \mathcal{H}(\boldsymbol{\lambda})|n(\boldsymbol{\lambda})\rangle|^2}{(\epsilon_m(\boldsymbol{\lambda}) - \epsilon_n(\boldsymbol{\lambda}))^2} \tag{9}$$

$$= \sum_{m\neq n} \frac{4|\Omega_{n\leftrightarrow m}(\boldsymbol{\lambda})|^2}{a_\mu^2 \omega_{n\leftrightarrow m}^2}. \tag{10}$$

For a two-level quantum system, the Rabi frequency of coherence transition is

$$\Omega_l(a_\mu) = 2|\Omega_{n\leftrightarrow m}(\boldsymbol{\lambda})| = a_\mu g_{\mu\mu}^{1/2}(\boldsymbol{\lambda})\omega_c, \tag{11}$$

where $\omega_c = \omega_{g\leftrightarrow e}$ represents the resonant frequency between the ground state and the excited state.

We proceed to consider general linear parametric modulation $\lambda_\mu(t) = \lambda_\mu^0 + a_\mu \sin(\omega t)$, $\lambda_\nu(t) = \lambda_\nu^0 + a_\nu \sin(\omega t)$ with $a_\mu \neq 0$ and $a_\nu \neq 0$. The amplitude of parametric modulation is small $|a_\mu|, |a_\nu| \ll 1$, thus the time-dependent Hamiltonian can be written as follows

$$\mathcal{H}[\boldsymbol{\lambda}(t)] = \mathcal{H}(\boldsymbol{\lambda}^0) + a_\mu \left[\partial_\mu \mathcal{H}(\boldsymbol{\lambda}^0)\right] \sin(\omega t) \tag{12}$$

$$+ a_\nu \left[\partial_\nu \mathcal{H}(\boldsymbol{\lambda}^0)\right] \sin(\omega t). \tag{13}$$

And the coherent transition Rabi frequency is

$$\Omega_{n\leftrightarrow m}(\boldsymbol{\lambda}) = \frac{1}{2}\langle m(\boldsymbol{\lambda})|a_\mu \partial_\mu \mathcal{H}(\boldsymbol{\lambda}) + a_\nu \partial_\nu \mathcal{H}(\boldsymbol{\lambda})|n(\boldsymbol{\lambda})\rangle. \tag{14}$$

Similarly, we can get the following relation between parametric modulation induced coherent transition and the Fubini-Study metric as

$$\sum_{m\neq n} \frac{4|\Omega_{n\leftrightarrow m}(\boldsymbol{\lambda})|^2}{\omega_{n\leftrightarrow m}^2} = a_\mu^2 g_{\mu\mu} + 2a_\mu a_\nu g_{\mu\nu} + a_\nu^2 g_{\nu\nu}. \tag{15}$$

In particular, for a two-level quantum system, the corresponding Rabi frequency of coherent transition $\Omega_l(a_\mu, a_\nu) = 2|\Omega_{n\leftrightarrow m}(\boldsymbol{\lambda})|$ is related to the Fubini-Study metric as follows

$$\Omega_l(a_\mu, a_\nu)^2/\omega_{g\leftrightarrow e}^2 = a_\mu^2 g_{\mu\mu} + 2a_\mu a_\nu g_{\mu\nu} + a_\nu^2 g_{\nu\nu}. \tag{16}$$

Therefore, one can extract the off-diagonal element of the Fubini-Study metric as follows

$$g_{\mu\nu} = \left[\Omega_l(a_\mu, a_\nu)^2 - \Omega_l(a_\mu, -a_\nu)^2\right] / \left(4a_\mu a_\nu \omega_{g\leftrightarrow e}^2\right). \tag{17}$$

The coherent response on the elliptical parametric modulation with $\lambda_\mu(t) = \lambda_\mu^0 + a_\mu \sin(\omega t)$, $\lambda_\nu(t) = \lambda_\nu^0 + a_\nu \cos(\omega t)$ can be analysed in a similar way. The corresponding time-dependent Hamiltonian can be written as follows

$$\mathcal{H}[\boldsymbol{\lambda}(t)] = \mathcal{H}(\boldsymbol{\lambda}^0) + a_\mu \left[\partial_\mu \mathcal{H}(\boldsymbol{\lambda}^0)\right] \sin(\omega t)$$

$$+ a_\nu \left[\partial_\nu \mathcal{H}(\boldsymbol{\lambda}^0)\right] \cos(\omega t). \tag{18}$$

The coherent transition Rabi frequency is

$$\Omega_{n\leftrightarrow m}(\boldsymbol{\lambda}) = \frac{1}{2}\langle m(\boldsymbol{\lambda})|a_\mu \partial_\mu \mathcal{H}(\boldsymbol{\lambda}) - i a_\nu \partial_\nu \mathcal{H}(\boldsymbol{\lambda})|n(\boldsymbol{\lambda})\rangle, \tag{19}$$

which is connected with the local Berry curvature as

$$\sum_{m\neq n} \frac{4|\Omega_{n\leftrightarrow m}(\boldsymbol{\lambda})|^2}{\omega_{n\leftrightarrow m}^2} = a_\mu^2 g_{\mu\mu} + a_\mu a_\nu \mathcal{F}_{\mu\nu} + a_\nu^2 g_{\nu\nu}. \tag{20}$$

For a two-level quantum system, we have the Rabi frequency of coherent transition $\Omega_c(a_\mu, a_\nu) = 2|\Omega_{n\leftrightarrow m}(\boldsymbol{\lambda})|$ is related to the Fubini-Study metric and the local Berry curvature as follows

$$\Omega_c(a_\mu, a_\nu)^2/\omega_{g\leftrightarrow e}^2 = a_\mu^2 g_{\mu\mu} + a_\mu a_\nu \mathcal{F}_{\mu\nu} + a_\nu^2 g_{\nu\nu}. \tag{21}$$

Therefore, one can measure the local Berry curvature in the following way

$$\mathcal{F}_{\mu\nu} = \left[\Omega_c(a_\mu, a_\nu)^2 - \Omega_c(a_\mu, -a_\nu)^2\right] / \left(2a_\mu a_\nu \omega_{g\leftrightarrow e}^2\right). \tag{22}$$

We remark that, for a discrete quantum system with more than two energy levels, the frequency shall be modulated in a larger range and a series of resonant transitions from the specific eigenstate to the other eigenstates shall be taken into account respectively. In the limit $a_\mu, a_\nu \ll 1$ where $\mu, \nu \in \{\lambda_1, \lambda_2, \cdots, \lambda_N\}$, each resonant transition can be approximated as a two-level system and the transition element $\langle m(\boldsymbol{\lambda})|\partial_\mu \mathcal{H}(\boldsymbol{\lambda})|n(\boldsymbol{\lambda})\rangle$ [see Eq.(3) in the main text] can be measured similarly.

A.2 Floquet analysis of coherent response on parametric modulation

The Hamiltonian of a two-level quantum system that we realize in the main text is given in Eq.(5). The system's response to periodic parametric modulation reveals information on quantum geometry. Here, we provide Floquet analysis for the example of linear parametric modulation $\theta(t) = \theta_0 + a_\theta \sin(\omega t)$, $\varphi(t) = \varphi_0 + a_\varphi \sin(\omega t)$ with $a_\theta = 0$ and $a_\varphi \neq 0$. In this case, the time-dependent Hamiltonian can be expanded as

$$\mathcal{H}(t) = \mathcal{H}_0(\theta_0, \varphi_0) + \sum_{n\neq 0} \mathcal{H}_n e^{in\omega t}, \tag{23}$$

with

$$\mathcal{H}_n = \frac{A}{2}\mathcal{J}_n(a_\varphi)\sin\theta_0 \begin{pmatrix} 0 & e^{-i\varphi_0} \\ (-1)^n e^{i\varphi_0} & 0 \end{pmatrix}, \tag{24}$$

where $\mathcal{J}_n$ is the n-th order Bessel function of the first kind. The eigenstates of the Hamiltonian $\mathcal{H}_0(\theta_0, \varphi_0)$ are

$$|\psi_1\rangle = \cos\frac{\theta_0}{2}|\uparrow\rangle + \sin\frac{\theta_0}{2}e^{i\varphi_0}|\downarrow\rangle, \tag{25}$$

$$|\psi_2\rangle = -\sin\frac{\theta_0}{2}e^{-i\varphi_0}|\uparrow\rangle + \cos\frac{\theta_0}{2}|\downarrow\rangle, \tag{26}$$

with the corresponding eigenenergy $\pm\frac{A}{2}$ respectively. Then one can rotate the above time-dependent Hamiltonian written in the basis $\{|\psi_1\rangle, |\psi_2\rangle\}$ as

$$\mathcal{H}'(t) = \frac{A}{2}\left(|\psi_1\rangle\langle\psi_1| - |\psi_2\rangle\langle\psi_2|\right) + \sum_{n\neq 0}\mathcal{H}'_n e^{in\omega t}, \tag{27}$$

where

$$\mathcal{H}'_{2n-1} = \frac{A}{2}\mathcal{J}_{2n-1}(a_\varphi)\sin\theta_0 \begin{pmatrix} 0 & e^{-i\varphi_0} \\ -e^{i\varphi_0} & 0 \end{pmatrix}, \tag{28}$$

$$\mathcal{H}'_{2n} = \frac{A}{2}\mathcal{J}_{2n}(a_\varphi)\sin\theta_0 \begin{pmatrix} \sin\theta_0 & \cos\theta_0 e^{-i\varphi_0} \\ \cos\theta_0 e^{i\varphi_0} & -\sin\theta_0 \end{pmatrix}. \tag{29}$$

Under rotating wave approximation, in the resonance case $\hbar\omega \cong A$, the higher order terms can be ignored and the above Hamiltonian can be simplified as

$$\begin{aligned}\mathcal{H}'(t) =& \frac{A}{2}\left(|\psi_1\rangle\langle\psi_1| - |\psi_2\rangle\langle\psi_2|\right) \\ &+ \frac{A}{2}\mathcal{J}_1(a_\varphi)\sin\theta_0 e^{i\omega t}\left(e^{-i\varphi_0}|\psi_1\rangle\langle\psi_2| - e^{i\varphi_0}|\psi_2\rangle\langle\psi_1|\right) + h.c.\end{aligned} \tag{30}$$

which leads to the following effective Hamiltonian as

$$\mathcal{H}_{\text{rot}} = \begin{pmatrix} \frac{A}{2} & -\frac{A}{2}\mathcal{J}_1(a_\varphi)\sin\theta_0 e^{i\varphi_0} \\ -\frac{A}{2}\mathcal{J}_1(a_\varphi)\sin\theta_0 e^{-i\varphi_0} & -\frac{A}{2} + \hbar\omega \end{pmatrix}. \tag{31}$$

Therefore, the Rabi frequency of coherent transition from the ground state to the excited state is

$$\Omega = \frac{A}{2}\mathcal{J}_1(a_\varphi)\sin\theta_0 = a_\varphi g_{\varphi\varphi}^{1/2}\omega_c/2. \tag{32}$$

We remark that the above Floquet analysis can be extended to general linear parametric modulation as well as elliptical parametric modulation.

A.3 Engineering of the effective Hamiltonian with parametric modulation

In the main text, we engineer a microwave driving field with amplitude, frequency and phase modulation [see Eq.(5)] acting on the NV center spin that leads to the following Hamiltonian as

$$\mathcal{H}(t) = \frac{\omega_0}{2}\sigma_z + A\sin\theta_t\cos\left[\omega_0 t - f(t) + \varphi_t\right]\sigma_x, \tag{33}$$

where θ_t and φ_t are periodically modulated parameters and $f(t)$ is a function which can be controlled in experiment. We first define the operator

$$\mathcal{K}(t) = \frac{\omega_0 t}{2}\sigma_z - \frac{A}{2}\int_0^t \cos\theta_\tau d\tau\sigma_z \equiv \mathcal{A}(t)\sigma_z. \tag{34}$$

The effective Hamiltonian in the rotating frame can be written as

$$\begin{aligned}\mathcal{H}_{\text{eff}} =& e^{i\mathcal{K}(t)}\mathcal{H}(t)e^{-i\mathcal{K}(t)} + i\Big(\frac{\partial e^{i\mathcal{K}(t)}}{\partial t}\Big)e^{-i\mathcal{K}(t)} \\ \cong& \frac{A}{2}\sin\theta_t\cos\left[A\int_0^t\cos\theta_\tau d\tau - f(t) + \varphi_t\right]\sigma_x \\ &+A\sin\theta_t\sin\left[A\int_0^t\cos\theta_\tau d\tau - f(t) + \varphi_t\right]\sigma_y \\ &+\frac{A}{2}\cos\theta_t\sigma_z,\end{aligned} \tag{35}$$

where we use rotating-wave approximation and neglect the fast oscillating terms with frequency $2\omega_0$. If we set the phase control function $f(t) = A\int_0^t\cos\theta_\tau d\tau$, the effective Hamiltonian can be simplified as

$$\mathcal{H}_{\text{eff}} \cong \frac{A}{2}\left[\cos\theta_t\sigma_z + \sin\theta_t\left(\cos\varphi_t\sigma_x + \sin\varphi_t\sigma_y\right)\right] \tag{36}$$

which is the desired Hamiltonian. In our experiments, we implement the phase control function $f(t)$ for $\theta_t = \theta_0 + a_\theta\sin(\omega t)$ as follows

$$\begin{aligned}f(t) =& A\int_0^t\cos\theta_\tau d\tau \\ =& A\int_0^t\cos\left[\theta_0 + a_\theta\sin(\omega\tau)\right]d\tau \\ =& A\int_0^t\Big\{\cos\theta_0\left[\mathcal{J}_0(a_\theta) + 2\sum_{n=1}^{\infty}\mathcal{J}_{2n}(a_\theta)\cos(2n\omega\tau)\right] \\ &- 2\sin\theta_0\sum_{n=0}^{\infty}\mathcal{J}_{2n+1}(a_\theta)\sin\left((2n+1)\omega\tau\right)\Big\} \\ \cong& A\cos\theta_0\mathcal{J}_0(a_\theta)t - \frac{4A\sin\theta_0}{\omega}\mathcal{J}_1(a_\theta)\sin^2(\omega t/2),\end{aligned} \tag{37}$$

where $\mathcal{J}_n(x)$ is the n-th order Bessel function of the first kind. The approximation in the last line is valid for the present scenarios with $a_\theta \ll 1$.

B. PROBING THE TOPOLOGY OF A TWO-LEVEL SYSTEM

B.1 Initial state preparation and verification

In our experiments, the first step is to prepare the NV center spin into the ground state of the unperturbed Hamiltonian $\mathcal{H}(\theta_0, \varphi_0)$ as follows

$$\mathcal{H}(\theta_0, \varphi_0) = \frac{A}{2}\begin{pmatrix} \cos\theta_0 & \sin\theta_0 e^{-i\varphi_0} \\ \sin\theta_0 e^{i\varphi_0} & -\cos\theta_0 \end{pmatrix}. \tag{38}$$

which is a superposition state $|\psi(0)\rangle = \cos\frac{\theta}{2}|-1\rangle + e^{i\varphi_0}\sin\frac{\theta}{2}|0\rangle$, see Eq.(41), in which we denote $|\uparrow\rangle \equiv |-1\rangle$ and $|\downarrow\rangle \equiv |0\rangle$. Such an initial state can be prepared by applying a resonant microwave driving field as

$$\mathcal{H}_p = \frac{\omega_0}{2}\sigma_z + \Omega\sin(\omega_0 t + \varphi)\sigma_x, \tag{39}$$

for time duration $t = \theta_0/\Omega$, where Ω is the Rabi frequency. If the initial state is not in the eigenstate of the Hamiltonian $\mathcal{H}(\theta_0, \varphi_0)$, it would cause oscillation that may blur the coherent oscillation arising from parametric modulation. In order to verify that we have indeed prepared the NV center spin into the right initial state, we perform experiments by engineering a microwave driving field corresponding to the Hamiltonian in Eq.(52). If the initial state is the ground state of $\mathcal{H}(\theta_0, \varphi_0)$, no transition to the excited state will be observed. In the experiments, we carefully tune the microwave pulse duration for the initial state preparation so that no transition to the excited state under the Hamiltonian $\mathcal{H}(\theta_0, \varphi_0)$ occurs, as shown in Fig.1.

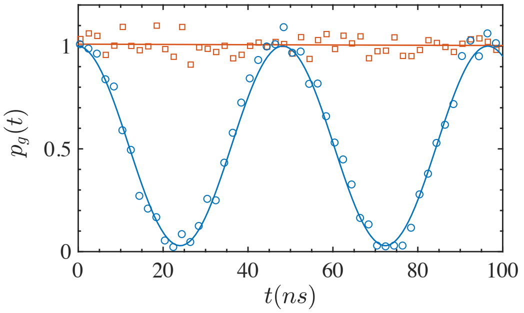

FIG. 1. Verification of initial state preparation. The state evolution from the initial state $|\psi(0)\rangle$, as governed by the Hamiltonian $\mathcal{H}(\theta_0, \varphi_0)$, is quantified by the fidelity $p_g(t) = |\langle\psi(t)|\psi(0)\rangle|^2$. The plot shows two initial states that are prepared by the microwave pulse $\mathcal{H}_i(t) = \Omega\sin(\omega_0 t + \varphi_0)\sigma_x$ with different time duration $t = 11.94$ ns (red, □) and $t = 0.04$ ns (blue, ∘).

B.2 Calibration of Rabi frequency and microwave amplitude

As we program parametric modulation in the waveform of AWG by specifying the microwave output amplitude, it is necessary to calibrate the relation between the microwave amplitude of AWG output and the Rabi frequency of the NV center spin. In

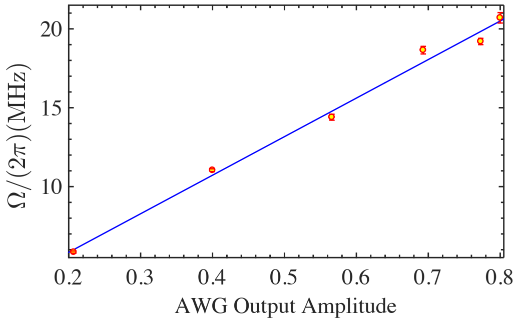

FIG. 2. Calibration of Rabi frequency and microwave amplitude. The measured Rabi frequency Ω of the NV center spin as a function of the output amplitude (in the unit of 500 mV [Vpp]) of arbitrary waveform generator while using the same microwave amplifying efficiency.

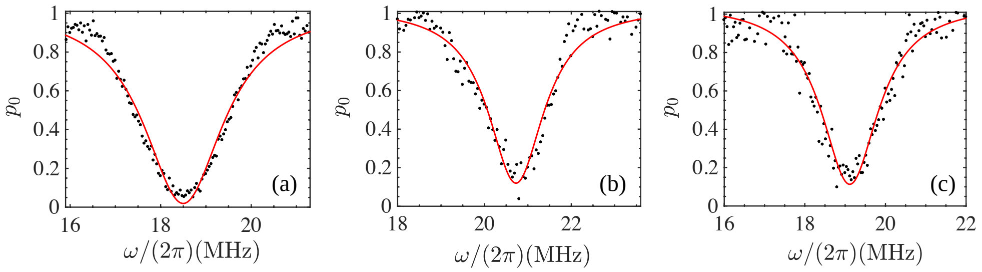

FIG. 3. Parametric modulation resonance measurement. The probability that the NV center spin remains in the ground state $|\psi(g)\rangle$ at time T as a function of the modulation frequency ω of (a) linear parametric modulation with $a_\theta = 0$, $a_\varphi = 0.08$, and $(\theta_0, \varphi_0) = (\pi/2, 0)$, $T = 450$ ns; (b) linear parametric modulation with $a_\theta = a_\varphi = 0.1$, and $(\theta_0, \varphi_0) = (3\pi/4, 0)$, $T = 400$ ns; (**c**) elliptical parametric modulation with $a_\theta = a_\varphi = 0.1$, and $(\theta_0, \varphi_0) = (\pi/6, 0)$, $T = 450$ ns.

Fig.2, we show one example of such a calibration, which shows that the Rabi frequency of the NV center spin scales linear with the AWG output amplitude for the parameter regime in which our experiments are performed. The microwave calibration is done for all the measurements.

B.3 Parametric modulation resonance measurement

In the parametric modulation resonance measurement experiments, we first prepare the NV center spin in the ground state $|\psi_0\rangle$ of the Hamiltonian $\mathcal{H}(\theta_0, \varphi_0)$ by a microwave pulse $\hat{n}(\varphi_0)|_{\theta_0}$. We then apply the engineered microwave driving field with different types of parametric modulation and fix the time duration T. By sweeping the parametric modulation frequency ω, we measure the probability $p_0(T)$ that the NV center spin remains in the ground state $|\psi_0\rangle$. When the parametric modulation frequency ω matches the transition frequency, we observe a resonance signal in the probability $p_0(T)$. In the main text, as shown in Fig.1(e), we provide an example of parametric modulation resonance measurement. In Fig.3, we show the parametric modulation resonance measurement data for the other types of parametric modulation.

B.4 Precise determination of parametric modulation resonance and oscillation frequency

After preparing the NV center spin in the ground state $|\psi_0\rangle$ of the Hamiltonian $\mathcal{H}(\theta_0, \varphi_0)$ by a microwave pulse $\hat{n}(\varphi_0)|_{\theta_0}$, we perform parametric modulation resonance measurement by sweeping the parametric modulation frequency ω, and measure the probability $p_0(T)$ that the NV center spin remains in the ground state $|\psi_g\rangle$, from which we can roughly determine the resonant frequency. The more accurate we determine the parametric modulation resonant frequency, the more precise we can extract the information on quantum geometry. Therefore, in the experiments, we optimise the parametric modulation frequency to achieve the best possible oscillation contrast in order to improve the accuracy in determining the Rabi frequency. Fig.4 shows coherent transition from the ground state to the excited state on parametric modulation with slightly different modulation frequency. It can be seen that when the resonant condition is better matched, the parametric modulation induced coherent oscillation demonstrates

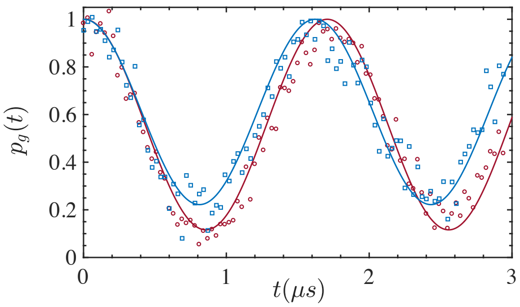

FIG. 4. Identification of exact resonance frequency. Coherent oscillation under linear parametric modulation with slightly different modulation frequency $\omega = (2\pi)20.3$ MHz (red, ○) and $\omega = (2\pi)20.28$ MHz (blue, □). The other parameters are: $\theta_0 = \pi/4$, $\varphi = 0$, $a_\theta = 0.1$ and $a_\varphi = 0.1$.

a higher contrast. We observe that the Rabi frequencies depend on the modulation amplitudes, but also, on the form of the parametric modulation, which is shown to represent direct signatures of the system's quantum geometry.

B.5 Detecting topological transition from measurement of quantum geometry

The topological transition can be detected in our experiment by extending the Hamiltonian into Eq.(12) with a tunable constant r that can be realised with an additional frequency detuning. For different value of r, the QGT of the Hamiltonian Eq.(12) can be calculated analytically as

$$g_{\theta\theta} = \frac{(1 + r\cos\theta)^2}{4(1 + r^2 + 2r\cos\theta)^2}, \tag{40}$$

$$g_{\varphi\varphi} = \frac{\sin^2\theta}{4(1 + r^2 + 2r\cos\theta)}, \tag{41}$$

$$g_{\theta\varphi} = 0, \tag{42}$$

$$\mathcal{F}_{\theta\varphi} = \frac{\sin\theta(1 + r\cos\theta)}{2(1 + r^2 + 2r\cos\theta)^{3/2}}. \tag{43}$$

With a finite value of r, all elements of the QGT can also be measured directly in our experiment using the similar method as described in the main text. In this case, the frequency of parametric modulation would depend on θ because the eigenenergies of Hamiltonian Eq.(12) are

$$E_\pm(r, \theta, \varphi) = \pm\frac{A}{2}\sqrt{1 + r^2 + 2r\cos\theta}, \tag{44}$$

which change with the value of θ. The NV center spin shall be initialised into the eigenstate

$$|\psi_g\rangle = \cos\frac{\theta'}{2}|-1\rangle + \sin\frac{\theta'}{2}e^{i\varphi}|0\rangle, \tag{45}$$

with

$$\theta' = \cos^{-1}\frac{\cos\theta + r}{\sqrt{1 + r^2 + 2r\cos\theta}} \tag{46}$$

which depends on the value of r.

When $r = 0$, we can look at the parameter space as a sphere S^2 embedded in $\mathbb{R}^3$, with a magnetic (Dirac) monopole at its origin. Changing the additional parameter r moves the location of this monopole away from the origin, and the topology of this parameter space is characterized by the number of monopoles inside the sphere. The number of monopoles is counted by the Chern number, which is the integral of the Berry curvature over the sphere [2]

$$C = \frac{1}{2\pi}\int_{S^2}\mathcal{F}_{\theta\varphi}d\theta d\varphi. \tag{47}$$

For a finite value of r, the distribution of Berry curvature $\mathcal{F}_{\theta\varphi}$ is not symmetric on both sides of $\theta = \pi/2$. When r approaches 1, the value of $\mathcal{F}_{\theta\varphi}$ has a singularity around $\theta = \pi$ which corresponds to a topological transition, where the monopole moves out of the sphere. In terms of the Chern number, the Chern number is $C = 1$ when $r < 1$, but $C = 0$ when $r > 1$.

C. QUANTUM GEOMETRY MEASUREMENT OF AN INTERACTING TWO-QUBIT SYSTEM

C.1 Description and characterisation of the system

Coupling the NV center to a neighboring nuclear spin allows us to explore the quantum geometry and topology of an interacting two-qubit system. In our experiment, this two-qubit system consists of the NV center electron spin and a ^{13}C nuclear spin located in the vicinity of the NV center. The nuclear spin of ^{14}N is polarised, such that $m_{I(N)} = +1$; in this setting, the total Hamiltonian of the NV electron spin (spin-1) and ^{13}C nuclear spin (spin-$\frac{1}{2}$) can be written as

$$\mathcal{H} = D_{gs}S_z^2 + \gamma_e B_\| S_z + \gamma_n B_\| I_z + A_z S_z \otimes I_z + A_x S_z \otimes I_x, \tag{48}$$

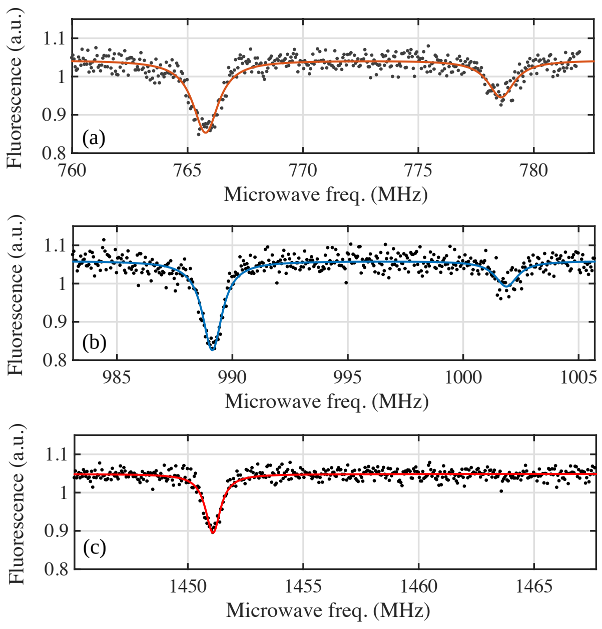

FIG. 5. Characterisation of spin-spin interaction. Pulsed optically detected magnetic resonance (pulsed ODMR) measurement with three different magnetic fields applying along the NV axis: $B_{\parallel}$ = 749.32 Gauss **(a)**, 669.64 Gauss **(b)**, 504.83 Gauss **(c)**. The two resonance dips corresponds to the transition frequencies ω_1 and ω_2 as in Eq.(49-50).

where S is a spin-1 operator and where I is the spin-$\frac{1}{2}$ operator; D_{gs} = 2.87GHz is the zero-field splitting, γ_e = 2.8MHz/G and γ_n = 1.07kHz/G is the electronic spin and nuclear spin gyromagnetic ratio, respectively.

We perform pulsed optically detected magnetic resonance (pulsed ODMR) measurement (see Fig.5) in order to determine the coupling strength (A_x and A_z) between the NV center electron spin and the ^{13}C nuclear spin. Following the Hamiltonian in Eq.(48), the transition frequencies are given by [3]

$$\omega_1 = \omega_s - \frac{1}{2}\sqrt{A_x^2 + (A_z - \gamma_c B_{\parallel})^2} - \frac{1}{2}\gamma_c B_{\parallel}, \tag{49}$$

$$\omega_2 = \omega_s + \frac{1}{2}\sqrt{A_x^2 + (A_z - \gamma_c B_{\parallel})^2} + \frac{1}{2}\gamma_c B_{\parallel}, \tag{50}$$

where $\omega_s = D_{gs} - \gamma_e B_{\parallel} - A_N^{hs}$, γ_e and γ_c represent the gyromagnetic ratio of the NV center electron spin and ^{13}C nuclear spin, respectively; A_N^{hs} = −2.16 MHz is the energy shift arising from the ^{14}N nuclear spin projection $m_{I(N)}$ = +1 associated with the NV center. The results for pulsed ODMR, as measured when applying three different magnetic fields along the NV axis, are shown in Fig.5. When combined with Eqs.(49)-(50), the measured transition frequencies ω_1 and ω_2 allow us to estimate the strength of the spin-spin interaction, and we obtain

$$A_x \approx 2.79\text{MHz} \qquad A_z \approx 11.832\text{MHz}. \tag{51}$$

We also point out that the pulsed ODMR measurement obtained by using a magnetic field of $B_{\parallel}$ = 505.13 Gauss along the NV axis [Fig.5(c)] only exhibits a single resonance; this result reflects the fact that the ^{13}C nuclear spin is effectively polarised at the excited-state level anti-crossing (ESLAC) point; see Ref. [4].

C.2 Details of topological property

In our experiment, we choose the NV center electronic spin manifold spanned by the spin sublevels $m_s = -1$ and $m_s = 0$ to encode the first qubit $|0\rangle \equiv |m_s = -1\rangle, |1\rangle \equiv |m_s = 0\rangle$. The nuclear spin of ^{13}C encodes the second qubit $|0\rangle \equiv \left|+\frac{1}{2}\right\rangle, |1\rangle \equiv \left|-\frac{1}{2}\right\rangle$. Based on this configuration, we write the Hamiltonian describing these two coupled qubits [Eq. (48)] as

$$\mathcal{H} = \frac{\omega_0}{2}\sigma_z + \left(\frac{\gamma_n B_{\parallel}}{2} - \frac{A_z}{4}\right)\tau_z - \frac{A_x}{4}\tau_x - \frac{A_z}{4}\sigma_z \otimes \tau_z - \frac{A_x}{4}\sigma_z \otimes \tau_x, \tag{52}$$

where $\boldsymbol{\sigma}$ and $\boldsymbol{\tau}$ are the Pauli operators associated with the first and second qubit; $\omega_0 = D_{gs} - \gamma_e B$ is the energy splitting between the states $m_s = 0$ and $m_s = -1$ of the NV center electron spin, which is controlled through the magnetic field $B_\parallel$ applied along the NV axis. Applying an additional microwave field

$$\mathcal{H}_{\rm mw} = \Omega_{\rm mw} \sin\theta(t) \cos\left(\omega_0 t - 2\Omega_{\rm mw}\int_0^t \cos\theta(\tau) d\tau + \varphi\right)\sigma_x, \tag{53}$$

one is able to rotate the electronic spin into an arbitrary direction; this protocol realizes the effective Hamiltonian in Eq.(13) of the main text. The Hamiltonian can be expressed in matrix form as

$$\mathcal{H}_{\rm rot}(\theta,\varphi) = \frac{1}{2}\begin{pmatrix} \Omega_{\rm mw}\cos\theta + \gamma_n B_\parallel - A_z & -A_x & \Omega_{\rm mw}\sin\theta e^{-i\varphi} & 0 \\ -A_x & \Omega_{\rm mw}\cos\theta - \gamma_n B_\parallel + A_z & 0 & \Omega_{\rm mw}\sin\theta e^{-i\varphi} \\ \Omega_{\rm mw}\sin\theta e^{i\varphi} & 0 & -\Omega_{\rm mw}\cos\theta + \gamma_n B_\parallel & 0 \\ 0 & \Omega_{\rm mw}\sin\theta e^{i\varphi} & 0 & -\Omega_{\rm mw}\cos\theta - \gamma_n B_\parallel \end{pmatrix}, \tag{54}$$

where we used the basis

$$|0\rangle|0\rangle = |-1\rangle_e |+1/2\rangle_n, |1\rangle|0\rangle = |0\rangle_e |+1/2\rangle_n, \tag{55}$$

$$|0\rangle|1\rangle = |-1\rangle_e |-1/2\rangle_n, |1\rangle|1\rangle = |0\rangle_e |-1/2\rangle_n. \tag{56}$$

Henceforth, we denote the eigenstates of the Hamiltonian in Eq. (54) as $|\Psi_1\rangle$, $|\Psi_2\rangle$, $|\Psi_3\rangle$, $|\Psi_4\rangle$, according to their ordered eigenenergies $\epsilon_1 < \epsilon_2 < \epsilon_3 < \epsilon_4$.

Importantly, we find that the topological properties of the NV center are enriched by the interaction with the neighboring nuclear spin ^{13}C. Indeed, in the single-qubit scenario, the Chern number of the ground state remains $C = 1$ for all values of the driving parameter $\Omega_{\rm mw}$. In contrast, in the interacting-qubit setting, the spin-spin interaction favors zero Chern numbers for all eigenstates; hence, tuning the drive parameter $\Omega_{\rm mw}$ allows one to drive topological phase transitions in this case. Specifically, the Chern number of the eigenstates $|\Psi_1\rangle$ and $|\Psi_3\rangle$ change from $C = 0$ to $C = 1$ after passing through the phase boundary corresponding to the critical value $\Omega_{\rm mw}^{(c_1)} = \frac{1}{2}\left[-\gamma_n B_\parallel + \sqrt{(\gamma_n B_\parallel - A_z)^2 + A_x^2}\right]$. Similarly, the Chern number of the eigenstates $|\Psi_2\rangle$ and $|\Psi_4\rangle$ change from $C = 0$ to $C = -1$. The significance of the QGT in interacting systems is highlighted by its connection with the quantum Fisher information, which characterizes the statistical distinguishability between eigenstates [5]. In particular,

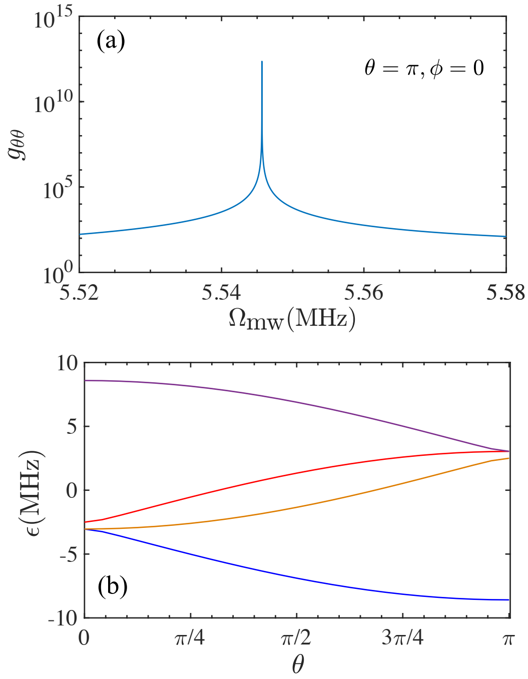

FIG. 6. (a) The Fubini-Study metric $g_{\theta\theta}$ (associated with the eigenstate $|\Psi_3\rangle$) as a function of $\Omega_{\rm mw}$ close to the critical value $\Omega_{\rm mw}^{(c_1)}$. (b) The eigenvalues as a function of the parameter θ for $\Omega_{\rm mw} = 5.5456$ MHz close to the critical value.

the singular behaviour of the QGT is expected to be associated with quantum critical phenomena [5]; we remind that the quantum metric is related to the generalized susceptibility, and hence to quantum fluctuations via the fluctuation-dissipation theorem [6, 7]. In Fig. 6(a), we plot the Fubini-Study metric $g_{\theta\theta}$ (associated with the eigenstate $|\Psi_3\rangle$), which clearly shows a singular behaviour. The singularity arises due the nearly degenerate eigenstates around the critical point, see Fig.6(b).

We also notice that the system features a second phase boundary at the critical value

$$\Omega_{\rm mw}^{(c_2)} = \frac{1}{2}\left[\gamma_n B_{\parallel} + \sqrt{(\gamma_n B_{\parallel} - A_z)^2 + A_x^2}\right], \tag{57}$$

where the eigenstates $|\Psi_2\rangle$ and $|\Psi_3\rangle$ undergo a topological phase transition associated with an exchange of the Chern number ($C = -1 \leftrightarrow C = 1$); we note that the topological properties of the eigenstates $|\Psi_1\rangle$ and $|\Psi_4\rangle$ are not modified through this process. We point out that in the regime $\Omega_{\rm mw} \gg \Omega_{\rm mw}^{(c_1)}$, the spin-spin interaction becomes less significant and we thus recover the topological properties of the two-level system, as we discussed in the case of two-level system.

C.3 Extracting the QGT elements for interacting qubits

1. Initial state preparation and verification

For arbitrary values of the parameters (θ, ϕ), the eigenstates of the interacting Hamiltonian in Eq.(12) are superposition states, which can be written in the form

$$\begin{aligned} |\Psi\rangle = \cos\vartheta \left(\cos\alpha_0 \left|-1\right\rangle_e + \sin\alpha_0 e^{i\beta_0} \left|0\right\rangle_e\right) \otimes \left|+1/2\right\rangle_c \\ + \sin\vartheta e^{i\eta} \left(\cos\alpha_1 \left|-1\right\rangle_e + \sin\alpha_1 e^{i\beta_1} \left|0\right\rangle_e\right) \otimes \left|-1/2\right\rangle_c . \end{aligned} \tag{58}$$

In our experiment, and without loss of generality, we have decided to measure the elements of the quantum geometry tensor associated with the eigenstate $|\Psi_3\rangle$. In fact, the initial state of the interacting spin system turns out to have a relatively high fidelity with the eigenstate $|\Psi_3\rangle$ when applying a magnetic field $B \sim 510$ Gauss (ESLAC) along the NV axis [see Fig.5(c)] together with a 532 nm polarizing laser pulse.

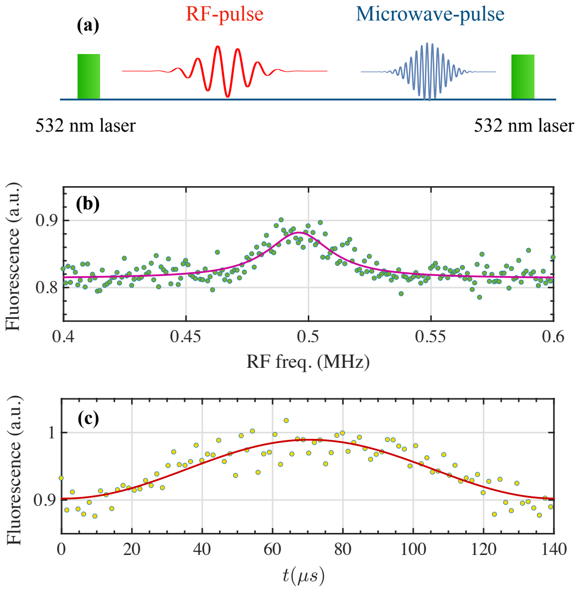

FIG. 7. Coherent manipulation of the ^{13}C nuclear spin. (a) Experimental sequence to determine the energy splitting of the ^{13}C nuclear spin and to observe Rabi oscillation: The first 532 nm laser pulse initialises the system and is followed by a RF-pulse acting on the ^{13}C nuclear spin; the subsequent selective microwave-pulse flips the NV center electron spin state conditioning on the ^{13}C nuclear spin state $|+1/2\rangle_c$ and the readout 532 nm laser pulse provides the information on the state population of the ^{13}C nuclear spin. (b) The resonance when sweeping the frequency of the RF-pulse indicates that the energy splitting of the ^{13}C nuclear spin (when the NV center spin is in the $m_s = 0$ state) is $\omega_c = 0.4961 \pm 0.0017$ MHz. (c) Rabi oscillation of the ^{13}C nuclear spin.

In order to achieve the high fidelity of the initial state preparation, one can first apply a radio-frequency pulse and prepare the ^{13}C nuclear spin into the following state

$$|\psi\rangle_c = \cos\vartheta\, |+1/2\rangle_c + \sin\vartheta e^{i\eta}\, |-1/2\rangle_c\,. \tag{59}$$

When the NV center electron spin is in the $m_s = 0$ state, the energy splitting of the ^{13}C nuclear spin is $\omega_c = \gamma_c B_\parallel$. As shown in Fig.7(a), we sweep the frequency of the radio-frequency pulse (while fixing the pulse length), and the subsequent microwave pulse selectively flips the NV center electron spin state conditioning on the ^{13}C nuclear spin state $|+1/2\rangle_c$. The resonance observed in Fig.7(b) indicates that the energy splitting of the ^{13}C nuclear spin is $\omega_c = 0.4961 \pm 0.0017$ MHz. By applying a radio-frequency field at such a resonant frequency, we then measure Rabi oscillations of the ^{13}C nuclear spin, see Fig.7(c). Subsequently, two selective microwave pulses that excite the electronic transition $|-1\rangle \leftrightarrow |0\rangle$ conditioning on the ^{13}C nuclear spin state $|+1/2\rangle_c$ and $|-1/2\rangle_c$, respectively, would prepare the whole system into the target eigenstate $|\Psi\rangle$ in the form given in Eq. (58). The protocol for the verification of the system's initial state is similar to that described in the above section B.1.

2. *Rabi oscillations induced by parametric modulation*

Our experiment aims to extract all the elements of the quantum geometric tensor (QGT) associated with an eigenstate of the interacting two-qubit setting; as explained above, we take this eigenstate to be $|\Psi_3\rangle$. To achieve such a goal, we engineer a microwave driving field associated with a specific parametric modulation, and we extract the relevant coupling matrix elements (generalized Rabi frequencies) entering the QGT through Rabi-oscillation measurements. A priori, the Rabi oscillations induced by parametric modulations involve all the eigenstates of the interacting-spin system: they take place between the state of reference $|\Psi_3\rangle$ and all the other three eigenstates $|\Psi_1\rangle$, $|\Psi_2\rangle$ and $|\Psi_4\rangle$. However, we find that the Rabi frequency related to the transition between $|\Psi_3\rangle$ and $|\Psi_1\rangle$ is an order of magnitude larger as compared to all other transitions (to $|\Psi_2\rangle$, $|\Psi_4\rangle$), when using the same experimental parameters. In this sense, the elements of the QGT related to the eigenstate $|\Psi_3\rangle$ are dominated by the generalized Rabi frequencies associated with the coherent transition between $|\Psi_3\rangle$ and $|\Psi_1\rangle$; we note that the contribution to the QGT scales as $\sim \Omega^2$ in terms of the generalized Rabi frequency Ω, see e.g. Eq.(24). In Fig.8, we plot the measured Rabi frequency Ω related to the coherent transition between the states $|\Psi_3\rangle$ and $|\Psi_1\rangle$, under linear and elliptical parametric modulations. These measurements allowed us to extract the complete quantum geometry tensor, which is displayed in Fig.5 (in the main text) together with theoretical predictions based on the Hamiltonian in Eq.(13).

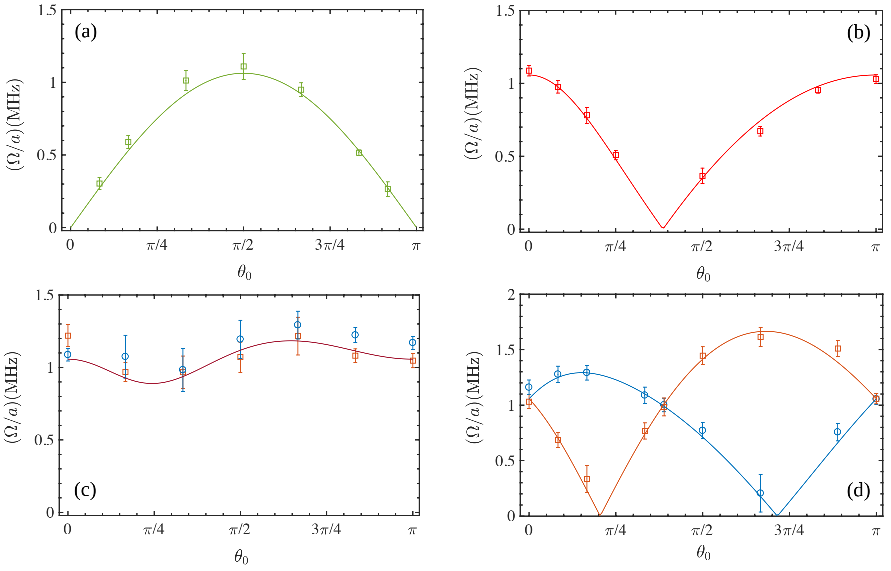

FIG. 8. Rabi frequency Ω of coherent transitions induced by parametric modulations, as a function of $\boldsymbol{\theta}$ for four types of parametric modulations: (a-b) Linear parametric modulation with $a_\phi = a$ and $a_\theta = 0$ (a); $a_\phi = 0$ and $a_\theta = a$ (b). (c) Linear parametric modulation with $a_\theta = a$ and $a_\phi = a$ (brown, □); $a_\theta = a$ and $a_\phi = -a$ (blue, ∘) (c). (d) Elliptical modulation with $a_\theta = a$ and $a_\phi = a$ (blue, ∘); $a_\theta = a$ and $a_\phi = -a$ (brown, □). The experiment parameters are: $\Omega_{\rm mw} = 2.13$ MHz. The curves represent the theoretical predictions.

[1] T. Ozawa and N. Goldman, *Extracting the quantum metric tensor through periodic driving*, Phys. Rev. B **97**, 201117(R) (2018).

[2] M. V. Berry, *Quantal phase factors accompanying adiabatic changes*, Proc. R. Soc. London, Ser. A **392**, 45-57 (1984).

[3] A. Dréau, J.-R. Maze, M. Lesik, J.-F. Roch, V. Jacques, *High-resolution spectroscopy of single NV defects coupled with nearby ^{13}C nuclear spins in diamond*, Phys. Rev. B **85**, 134107 (2012).

[4] V. Jacques, P. Neumann, J. Beck, M. Markham, D. Twitchen, J. Meijer, F. Kaiser, G. Balasubramanian, F. Jelezko, and J. Wrachtrup, *Dynamic Polarization of Single Nuclear Spins by Optical Pumping of Nitrogen-Vacancy Color Centers in Diamond at Room Temperature*, Phys. Rev. Lett. **102**, 057403 (2009).

[5] P. Zanardi, P. Giorda, M. Cozzini, *Information-theoretic differential geometry of quantum phase transitions*, Phys. Rev. Lett. **99**, 100603 (2007).

[6] M. Kolodrubetz, D. Sels, P. Mehta, A. Polkovnikov, *Geometry and non-adiabatic response in quantum and classical system*, Phys. Rep. **697**, 1-87 (2017).

[7] T. Ozawa and N. Goldman, *Probing localization and quantum geometry by spectroscopy*, arXiv:1904.11764.
